# COVID-19 and its effects on food producers: panel data evidence from Burkina Faso

**DOI:** 10.1186/s40795-024-00942-x

**Published:** 2024-10-08

**Authors:** Thomas Druetz, Sara Brenes-Garita, Frank Bicaba, Cheick Tiendrebeogo, Abel Bicaba

**Affiliations:** 1 Department of Tropical Medicine and Infectious Disease, School of Public Health and Tropical Medicine, Tulane University, Suite 2300, 1440 Canal St., New Orleans, LA 70112 USA; 2 School of Public Health, University of Montreal, CP 6128, Succursale Centre Ville, Montreal, QC H3C 3J7 Canada; 3 Centre de recherche en santé publique, 7101 Park av, Montreal, QC H3N 1X9 Canada; 4 Société d’études et de recherches en santé publique, Rue 28.247, Secteur 44 Dassasgho, Ouagadougou, Burkina Faso

**Keywords:** Food insecurity, COVID-19, Burkina Faso, Pandemic, Food production, Livestock

## Abstract

**Introduction:**

Burkina Faso implemented stringent measures in response to the COVID-19 pandemic that profoundly affected its economy and might have exacerbated food insecurity. While prior studies have assessed the impact of these measures on consumers, there is a dearth of evidence of its effects on food producers in Sub-Saharan Africa. This study aims (i) to evaluate the repercussions of COVID-19 on the possession of food production assets and on the number of livestock owned; and (ii) to determine the correlation between the food insecurity experience scale (FIES) score, ownership of these assets, and the number of livestock owned.

**Methods:**

This study employs a pre-post comparison design in two panels of randomly selected households in Burkina Faso. While Panel A was constituted of 384 households predominantly (76%) living in rural areas, Panel B comprised 504 households, only half of which (51%) lived in rural areas. All households were visited twice: in July 2019 and February 2021, for Panel A, and in February 2020 and February 2021, for Panel B. Panel B was added to the study before the pandemic thanks to additional funding; the timing of the survey was harmonized in both panels for the second round. Regression models were used with fixed effects at the household level, controlling for potential time-invariant confounding variables, and correlation coefficients between possession of production assets or number of livestock and FIES score were measured.

**Results:**

Our findings indicate that the possession of some assets in Panel A (cart, livestock, bicycle, watch) was significantly reduced during the pandemic, as was the herd sizes among livestock-owning households in both panels. Households with fewer production assets and number of livestock were more likely to experience food insecurity.

**Conclusion:**

This study underscores the vulnerability of rural households in Burkina Faso to the economic disruptions caused by the COVID-19 pandemic. Addressing the challenges faced by farming and livestock-owning households is crucial for mitigating food insecurity and improving resilience in the face of ongoing crises.

**Supplementary Information:**

The online version contains supplementary material available at 10.1186/s40795-024-00942-x.

## Background

Burkina Faso is a landlocked Sahelian country with a population of 21 million. Poverty has been gradually decreasing in recent years, but approximately 40% of the population remains under the national poverty line, and the country is ranked 196th out of 203 on the human development index. Historically, around 90% of the population has been living in rural areas, but this figure has steadily declined in recent decades and was closer to 70% in 2023 [1]. About 80% of the total workforce remains primarily employed in agriculture, among which 70% practice livestock husbandry [2, 3].

The overall situation for the rural population has been deteriorating in recent years. Since 2015, Burkina Faso has seen an exponential increase in attacks on civilians, particularly by terrorist groups [4]. This growing climate of insecurity mainly affects rural areas and revives local tensions, particularly between ethnic groups. It also makes trade and travel more difficult, and harms economic activity in general. Compounding these issues is climate change, which hampers agricultural production, increases pressure on farmland, and fuels conflict between farmers and herders [5, 6].

The COVID-19 pandemic therefore occurred in an already precarious context. Burkina Faso has seen three waves of COVID-19: March - May 2020, December 2020 - March 2021, September 2021 - February 2022. While the total morbidity and mortality burden (respectively ∼22,000 cases and ∼400 deaths) might now seem moderate compared to other countries, the situation was highly concerning during the first wave, when Burkina Faso had the highest mortality rate in West Africa [7, 8]. Anticipating an out-of-control epidemic, the authorities took health security measures as soon as March 2020 to contain the COVID-19 pandemic. Some of these measures profoundly affected economic life and work in the general population, particularly a national curfew, the closure of public markets and nonessential activities, the closure of international borders, the banning of gatherings of > 50 people, the restrictions on road travel between regions, and (in the largest cities) quarantine [7, 9].

Mitigation measures in themselves quickly became a source of concern. Faced with the threat of their own measures having harmful consequences on the economy, poverty, and on population health, the authorities rapidly set up a multi-sectoral committee to reorient the strategic response plan [7]. For this reason, most of these public health measures taken during the first wave were implemented only for a few months, between March – September 2020. Although short-lived, their impact on the national economy was far-reaching. For instance, economic growth fell by 4% between 2019 and 2020^5^.

This situation is not unique to Burkina Faso. Soon after the worldwide imposition of public health measures against the pandemic, experts drew attention to their potentially harmful effects on global agricultural production, trade, food insecurity, and poverty [10, 11]. These effects could be fueled by a number of simultaneous mechanisms, including reduced ability to buy, sell, or transport food; interruptions in agricultural production; supply-chain disruptions; losses in income-generating activities; and discontinuance of programs to mitigate acute malnutrition in children, including school meals [12]. Concerns were even more pronounced for low- and middle-income countries (LMICs) due to their limited resilience to maintaining their population’s purchasing power in times of economic or financial crises [13, 14].

Empirical evidence has since confirmed these predictions. Systematic reviews have shown that the pandemic has escalated household impoverishment and food insecurity in most settings, although the effects vary according to factors like the extent of the protective measures, support from governments, and individual households’ socioeconomic vulnerability [15–21]. In West Africa, deleterious effects have appeared in both rural and urban areas in the short term (> 3 months) to medium term (3–12 months) after the onset of the pandemic [22–26].

A recent scoping review noted that most of the studies conducted in the region have examined the effects on consumers in the food chain rather than producers [24]. There is little evidence of the impact of COVID-19 on poverty or food insecurity among farmers and livestock owners in West Africa in general and in Burkina Faso in particular [27]. This is concerning, since they are particularly vulnerable to food insecurity compared to households in urban areas or those living in rural areas with non-farm income [28, 29].

Using data from a panel study conducted in rural and semi-rural Burkina Faso (before and during the pandemic), this study sets out to (1) evaluate the repercussions of the COVID-19 on the possession of food production assets and on the number of livestock owned; and (2) assess household FIES scores during the pandemic and their correlation with ownership of assets and number of livestock owned.

## Methods

### Study design

The study is a natural experiment based on two panels of households that were constituted for the purpose of another study [9]. Panel A was constituted of 384 households living predominantly (76%) in rural areas. Panel B comprised 504 households, only half of which (51%) lived in rural areas. All households were visited twice: in July 2019 and February 2021, for Panel A, and in February 2020 and February 2021, for Panel B. The pre-pandemic survey in Panel B occurred later (February 2020) than in Panel A (July 2019) because new funding allowed to extend the study to a second area. Subsequently, the onset of the pandemic disrupted the survey schedule, and it was decided to harmonize the second round in both panels one year after the start of the pandemic, i.e., in February 2021. The study area covers eight districts (four per panel), all located > 100 km from the capital city of Ouagadougou. These districts mainly cover rural areas, although they also include semi-rural areas and small cities. Due to the deteriorating security situation in the country, the districts selected for sampling Panel B households were located more in the south-west of Burkina Faso. In this area, agriculture is somewhat less prevalent, and the population is more diverse in terms of ethnic, religious and socio-economic composition.

For the primary objective of evaluating the effects of the COVID-19 pandemic on household possession of food production assets, we designed a pre-post study using the repeated measures in each household. For the secondary objective of determining the correlation between asset ownership and FIES score (0–8, where 8 is most severe), the analysis used only the data collected during the pandemic in February 2021. Detailed information about the households can be found elsewhere [30].

### Recruitment and survey procedures

Recruitment procedures used a stratified two-stage random sampling method, as detailed elsewhere [31]. Briefly, the sampling procedures were derived from those used by the Demographic and Health Surveys (DHS) [32]. A sample size of maximum 700 households was targeted for each panel. Households without any women of reproductive age (15–49 years old) were excluded. After being recruited and surveyed at baseline, each household was systematically surveyed 12–18 months later, during the post-pandemic round. The panels were closed to enrollment; there were no replacements for households lost during follow-up.

A questionnaire was administered in each household to document its composition and its ownership (yes/no) of seven socioeconomic assets useful for food production: cart, livestock, land, plough, bicycle, motorbike, watch (“production assets” in the text). Among households that responded that they had livestock, a complete enumeration was carried out (“number of livestock” or “herd size” in the text). All questions were extracted from standardized DHS instruments, to which was added the FIES module, developed by the Food and Agriculture Organization of the United Nations [33]. The FIES consists of eight questions regarding the household’s access to adequate food. They focus on self-reported food-related experiences associated with difficulties in accessing food due to resource constraints. The survey was administered by experienced research assistants, over 80% of whom had participated in at least two survey rounds.

### Analyses

An aggregated score for the FIES was calculated for each household following an established procedure [15, 33]. Each positive answer of the 8 questions was given a score of 1, and 0 otherwise, so that the aggregated score ranged between 0 (no experience of food insecurity) to 8 (maximal experience of food insecurity). Somers’ D coefficients (commonly used to conservatively measure the association between a binary and an ordinal variable) were calculated to assess the association between the FIES score for each household and whether it had access to production assets (yes/no). Among livestock-owning households, Kendall’s coefficients (commonly used to measure the association between two ordinal variables) were computed to assess the correlation between the FIES score and the number of animals owned for each category of livestock.

The repercussions of the COVID-19 pandemic on the ownership of production assets and on the number of livestock owned was assessed individually for each category of assets. Regression models with a logistic (for binary outcomes) or negative binomial (for count outcomes) distribution were fitted using fixed effects at the household level. Fixed effects in longitudinal panels allow for control for any confounding variable that is stable over time within the household [34]. All models included the year of the survey (2019/2020 or 2021) to isolate pre-post pandemic change, and the size of the household as a potential time-varying confounding variable. Households lost to follow-up were excluded from the analysis. All analyses were performed using Stata version 14.0 software (StataCorp LLC, College Station, Texas).

### Ethics

All participants recruited pre-pandemic provided written informed consent. As suggested and approved by the ethics committees in Burkina Faso and in Canada, all participants recruited in 2021 provided informed consent verbally in order to reduce the risk of COVID-19 transmission. All study procedures were approved by the Health Sciences Research Ethics Committee at University of Montreal (Certificate #CERSES-20-146-D) and by the Health Research Ethics Committee in Burkina Faso (Deliberation #2018-6-075). The study was performed in accordance with the Declaration of Helsinki.

## Results

Among the 1,181 households that were surveyed before the pandemic, 293 (25%) were lost to follow-up. Among the households that we could find and visit again (*n* = 888), none refused to participate in the second survey. Compared to households visited twice, those lost to follow-up had significantly lower ownership of production assets and of the number of livestock in the pre-pandemic survey (Appendix 1), meaning that our results may underrepresent temporary households or those in the most precarious situations. The 888 households visited twice were members either of Panel A (*n* = 384, 43%) or of Panel B (*n* = 504, 57%). Most households were located in rural areas, although the proportion was higher in Panel A than in Panel B (76% vs. 51%) (Table 1).

**Table 1:** Socio-demographic characteristics of the participating households, by panel

	Panel A	Panel B	Difference
Number of households	384	504	
Rural	286 (0.76)	259 (0.51)	< 0.001
Household size	10	8	< 0.001
Muslim	327 (0.85)	306 (0.61)	< 0.001
Owns livestock	316 (0.82)	384 (0.76)	< 0.05
Head went to primary school	127 (0.35)	185 (0.42)	< 0.05
Socioeconomic status			0.141
Poorest	88 (0.22)	87 (0.17)	
Medium poor	77 (0.2)	105 (0.21)	
Medium	82 (0.21)	98 (0.19)	
Medium rich	78 (0.20)	112 (0.22)	
Richest	58 (0.15)	99 (0.2)	
Household is polygamous	166 (0.43)	164 (0.32)	< 0.01

### Food insecurity experience scale

**Fig. 1:**
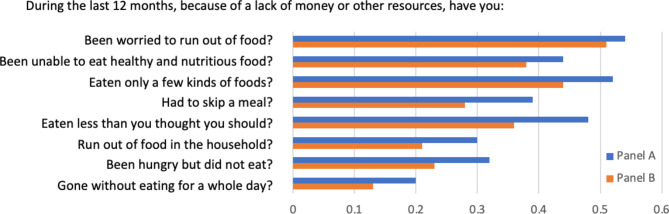
Households’ experience of food insecurity (in %, by panel)

In 2021, the experience of food insecurity was high among the two panels, with > 50% of all household respondents citing food as a concern (Fig. 1); however, the experience of food insecurity was significantly (*p* < 0.05) more common in Panel A than in Panel B for most of the 8 indicators. On a scale of 0 (no insecurity) to 8 (maximal insecurity), the aggregated total score reached 3.19 (Panel A) and 2.54 (Panel B) on average. The significant difference (δ = 0.65, *p*-value < 0.001) indicates a more dire situation regarding food insecurity in Panel A than in Panel B. Moderate to severe food insecurity (FIES score 4–8) concerned 43% in Panel A vs. 33% in Panel B (*p*-value < 0.005).

### Effects of COVID-19 pandemic on households’ production assets

At the baseline, possession of the production assets varied between the two panels. Panel A presented a more rural profile than Panel B, with significantly higher ownership levels of land, livestock, ploughs, and carts (Appendix 2). Possession of assets associated with economic productivity in general (i.e., watches, vehicles and motorbikes) was significantly higher in Panel B than in Panel A.

**Fig. 2:**
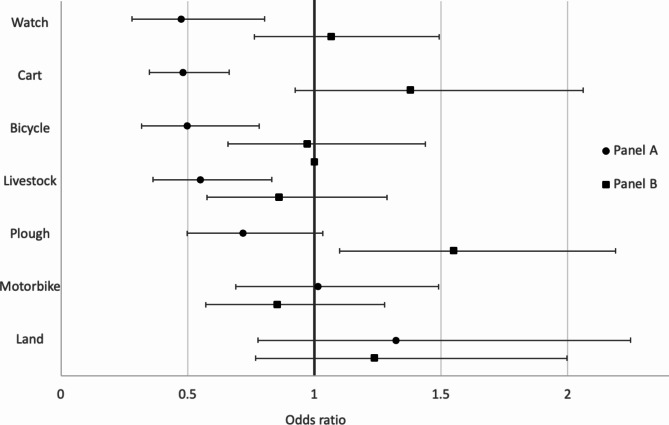
Effects of the pandemic on the ownership of key production assets (by panel). Effects estimates are derived from logistic regression models with fixed effects at the household level and robust variance estimators. Effects are displayed with their 95% confidence intervals

After adjusting for family size, the models with fixed effects at the household level suggest no significant pre-post pandemic change for any of the production assets in Panel B (Fig. 2). In Panel A, models indicate significant reduction in the odds of owning watches (OR_A_ = 0.47, 95% CI [0.28–0.8]), livestock (OR_A_ = 0.55, 95% CI [0.36–0.83]), carts (OR_A_ = 0.48, 95% CI [0.35–0.66]) and bicycles (OR_A_ = 0.50, 95% CI [0.31–0.78]).

### Effects of COVID-19 pandemic on the number of livestock owned

The average number of goats and sheep per livestock-owning household were similar at baseline between the two panels. Farmers with livestock in Panel B had significantly more cattle, cows and poultry, but fewer horses, than in Panel A (Appendix 3).

**Fig. 3:**
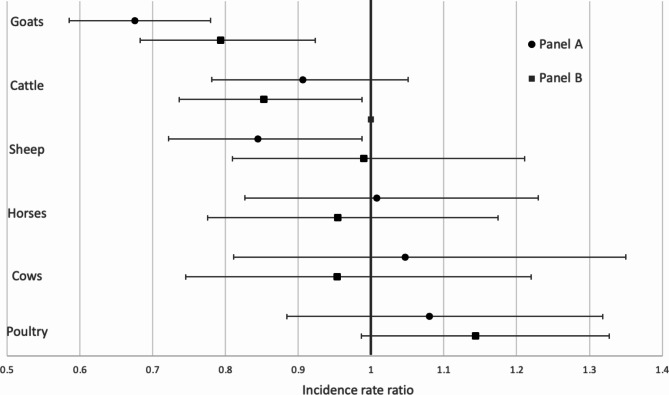
Effects of the pandemic on the number of livestock owned by pastoralists (by panel). Effects estimates are derived from negative binomial regression models with fixed effects at the household level and robust variance estimators. Effects are displayed with their 95% confidence intervals

Models with fixed effects at the household level indicate that pre-post pandemic changes were not statistically significant for poultry, cows, or horses. Significant decreases in the possession of goats were recorded for both panels (IRR_A_ = 0.68, 95% CI [0.59–0.78] and IRR_B_ = 0.79, 95% CI [0.68–0.92]) (Fig. 3). In panel A, there was a significant reduction in sheep ownership (IRR_A_ = 0.84, 95% CI [0.72–0.99]), while the possession of cattle significantly decreased in panel B (IRR_B_ = 0.85, 95% CI [0.74–0.99]).

### Correlation between production assets and FIES score

**Fig. 4:**
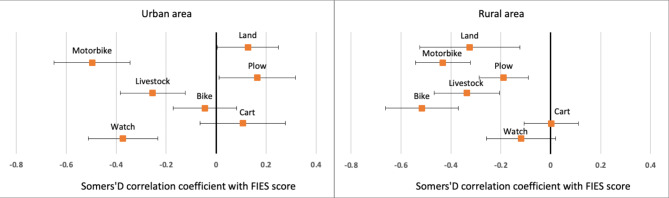
Somer’s D correlation coefficient between the household FIES score and ownership of key production assets during the pandemic (by living area). Coefficients are displayed with their 95% confidence interval

Somer’s D correlation coefficients between ownership of production assets (yes/no) and household FIES score were computed by type of living area (urban or rural) and individually for each type of asset. In urban areas, the possession of motorbikes, watches, and livestock was associated with a reduction in food insecurity experience, while owning agricultural assets or lands was positively correlated with food insecurity (Fig. 4). In rural areas, the possession of all types of assets except for carts and watches was associated with a significant reduction in FIES score. The assets mostly correlated with food insecurity were motorbikes, bicycles, land, and livestock.

### Correlation between livestock and FIES score

**Fig. 5:**
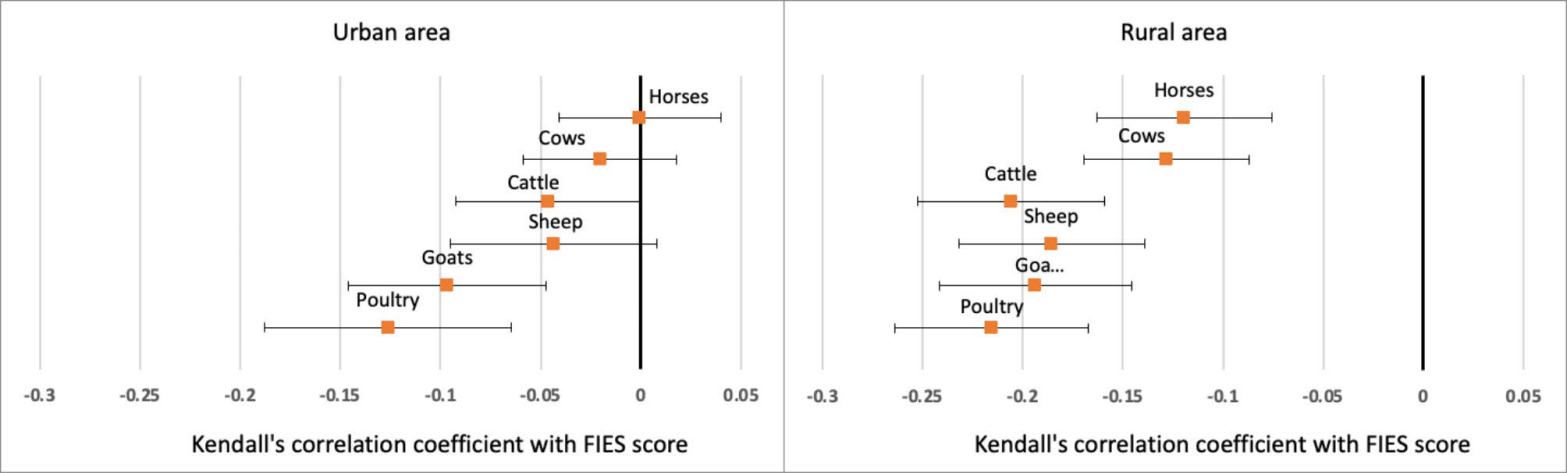
Kendall’s correlation coefficient between the household FIES score and number of livestock owned by pastoralist during the pandemic (by living area). Coefficients are displayed with their 95% confidence interval

Owning more livestock, regardless of type, was always correlated with less food insecurity, although the correlation was larger and more significant in rural compared to urban areas (Fig. 5). The strongest individual correlations were with the number of poultry, cattle, goats, and sheep owned.

## Discussion

This study corroborates the hypothesis that, during the pandemic, there was a significant loss of some production assets and a reduction in herd size in rural and semi-rural households in Burkina Faso. The effects on production assets were only statistically significant in one of our panels (Panel A), surveyed twice at 18-month intervals, and not in the other one surveyed 12 months apart (Panel B). Although the hypothesis cannot be ruled out, it is unlikely that seasonality played a significant role in this difference in effects. Indeed, the pre-pandemic survey in Panel A took place in July, during the lean season, i.e., the time of the year households are already the most deprived and vulnerable. The post-pandemic survey took place after the harvest season, when households are more affluent. Therefore, if seasonality had had an influence, it would have counteracted the effect of the pandemic, and our effect estimates would have been more limited in Panel A than in Panel B – the opposite of what we observed. Rather, our analyses suggest that the difference can be explained by the contrasting profiles of the two panels, notably the predominant rurality of Panel A. When restricting the analyses to livestock-owning households, a decrease in the number of goats was observed in both panels. A decline in the number of sheep (Panel A) and cattle (Panel B) possessed by the households was also observed. These three ruminant livestock are the most widely used for pastoralism in Burkina Faso [35].

Although other studies have examined the effects of COVID-19 on food systems in Africa, ours is among the first to look upstream at production capacity, rather than downstream at consumption capacity [24]. Our results are in line with a study conducted in Tanzania and South Africa that showed that farmers had to sell some of their livestock for short-term financial relief [36]. This coping strategy was triggered not only by the COVID-19 restrictions but also because of limited access to capital. This is consistent with the reduction in herd sizes among livestock farmers observed in Kenya and attributed to the pandemic [37]. Similar livestock asset depletion was directly observed in Chad and Uganda [38, 39] and self-reported by households in six African countries [40]. While necessary in the short term, this type of destocking can severely reduce pastoralists’ resilience to future shocks and can lead to a vicious cycle of poverty.

Our analyses also show that, in rural areas, the experience of food insecurity is negatively correlated to the possession of all types of production assets (other than watches and carts) or number of livestock owned. Interestingly, in urban areas, the possession of agricultural production assets like land and plows is a risk factor of increased food insecurity. This is in line with other studies of living conditions in informal settlements surrounding cities (locally known as “non-loti” neighborhoods) showing that agricultural households living in these peri-urban areas are particularly precarious compared to non-agricultural households [41, 42].

By showing that COVID-19 led to losses in production assets and in the number of livestock owned, and by establishing the correlation between owning such assets and livestock and food insecurity, this study contributes to establishing how COVID-19 has affected food insecurity in Burkina Faso, particularly in rural areas. While some have suggested that short-term effects were more important in urban areas, our results indicate that medium-term effects on poverty and food insecurity were particularly significant in rural households [15].

In our panel, 68% of households suffer from mild-severe food insecurity, higher than the 55% prevalence measured at the same time by phone in ∼ 2400 Burkinabè households using the same survey instrument [15]. Measurement issues (underestimations associated with phone-based surveys [43]) or selection bias are plausible explanations for this gap, since neither study sought to estimate food insecurity in a sample representative of the total population [34]. Still, the prevalence found in our cohort is worrisome: 42% of rural households were experiencing moderate food insecurity and 9–13% severe food insecurity. The pandemic exacerbated the difficulties already faced by small-scale, subsistence farmers, including terrorist attacks, growing financial insecurity, and severe effects of climate change [4, 44]. The pandemic has illustrated the low resilience of rural households and their difficulty absorbing shocks; simultaneously, models predict that Burkina Faso will be one of the African countries most affected by environmental crises, including episodes of drought, heat waves, and torrential rain, with catastrophic repercussions for agriculture [45, 46]. This puts rural households in a situation of extreme vulnerability with potentially detrimental consequences for public health; particularly child undernutrition, for which Burkina Faso already has one of the highest rates in the world [47–50].

### Limitations

This study used a strong quasi-experimental design (a pre-post comparison with fixed effects at the household level) to evaluate the repercussions of COVID-19; however, the absence of a control group limits our ability to attribute results to the pandemic [51]. Other isolated factors or events may have contributed to the observed losses in assets and reductions in herd sizes. Secondly, our results are limited to a sub-sample of households that our team visited twice and therefore are not representative of the entire population, although analyses show that households lost to follow-up were less affluent, meaning the selection bias would have reduced the results towards a null effect (also called “conservative bias” since the true effect should be larger than the one actually measured) [52].

## Conclusion

This study shows that, one year after its onset, the COVID-19 pandemic had some negative repercussions on the wealth of rural populations in Burkina Faso, particularly farmers and livestock-owning households. We found decreases in household ownership of production assets, and lower herd sizes of the most common livestock used for pastoralism. It also implies that, in rural areas, households owning fewer of these assets or livestock are more likely to experience food insecurity. With a prevalence of moderate-to-severe food insecurity of 42%, our study draws attention to the profound vulnerability of Burkinabè households in rural areas, particularly in view of the polycrisis that prevails in the region. Reducing child malnutrition, food insecurity, and health inequities requires specifically targeting rural households to improve their resilience.

## Electronic supplementary material

Below is the link to the electronic supplementary material.

Supplementary Material 1

Supplementary Material 2

Supplementary Material 3

## Data Availability

The datasets used and/or analysed during the current study are available from the corresponding author on reasonable request.
