# Supplementary material for: COVID-19 and its effects on food producers: panel data evidence from Burkina Faso
Source: BMC Nutr. 2024 Oct 8;10:132. doi: 10.1186/s40795-024-00942-x (PMC11462756; doi:10.1186/s40795-024-00942-x)
Supplement: Supplementary file 1 — Supplementary Material 1 [file 40795_2024_942_MOESM1_ESM.docx]

Appendix 1

1. **Test of difference between households surveyed once (lost to follow up) vs. twice in regard to the owning of production assets.**

. bysort ds_cat: tab Q118Z_1 temp if year!=2021, chi2

-------------------------------------------------------------------------------------------------------

-> ds_cat = batch_2019

| temp

Watch | 0 1 | Total

-----------+----------------------+----------

0 | 76 315 | 391

1 | 24 69 | 93

-----------+----------------------+----------

Total | 100 384 | 484

Pearson chi2(1) = 1.8592 Pr = 0.173

-------------------------------------------------------------------------------------------------------

-> ds_cat = batch_2020

| temp

Watch | 0 1 | Total

-----------+----------------------+----------

0 | 131 375 | 506

1 | 62 129 | 191

-----------+----------------------+----------

Total | 193 504 | 697

Pearson chi2(1) = 2.9905 Pr = 0.084

. bysort ds_cat: tab Q118Z_2 temp if year!=2021, chi2

-------------------------------------------------------------------------------------------------------

-> ds_cat = batch_2019

| temp

Plough | 0 1 | Total

-----------+----------------------+----------

0 | 68 195 | 263

1 | 32 189 | 221

-----------+----------------------+----------

Total | 100 384 | 484

Pearson chi2(1) = 9.4805 Pr = 0.002

-------------------------------------------------------------------------------------------------------

-> ds_cat = batch_2020

| temp

Plough | 0 1 | Total

-----------+----------------------+----------

0 | 139 321 | 460

1 | 54 183 | 237

-----------+----------------------+----------

Total | 193 504 | 697

Pearson chi2(1) = 4.3155 Pr = 0.038

. bysort ds_cat: tab Q118Z_3 temp if year!=2021, chi2

-------------------------------------------------------------------------------------------------------

-> ds_cat = batch_2019

Une | temp

bicyclette | 0 1 | Total

-----------+----------------------+----------

0 | 19 61 | 80

1 | 81 323 | 404

-----------+----------------------+----------

Total | 100 384 | 484

Pearson chi2(1) = 0.5578 Pr = 0.455

-------------------------------------------------------------------------------------------------------

-> ds_cat = batch_2020

Une | temp

bicyclette | 0 1 | Total

-----------+----------------------+----------

0 | 55 97 | 152

1 | 138 407 | 545

-----------+----------------------+----------

Total | 193 504 | 697

Pearson chi2(1) = 7.0048 Pr = 0.008

. bysort ds_cat: tab Q118Z_6 temp if year!=2021, chi2

-------------------------------------------------------------------------------------------------------

-> ds_cat = batch_2019

Une |

charrette |

tirée par | temp

un animal | 0 1 | Total

-----------+----------------------+----------

0 | 49 163 | 212

1 | 51 221 | 272

-----------+----------------------+----------

Total | 100 384 | 484

Pearson chi2(1) = 1.3837 Pr = 0.239

-------------------------------------------------------------------------------------------------------

-> ds_cat = batch_2020

Une |

charrette |

tirée par | temp

un animal | 0 1 | Total

-----------+----------------------+----------

0 | 152 357 | 509

1 | 41 147 | 188

-----------+----------------------+----------

Total | 193 504 | 697

Pearson chi2(1) = 4.4477 Pr = 0.035

. bysort ds_cat: tab Q118Z_8 temp if year!=2021, chi2

-------------------------------------------------------------------------------------------------------

-> ds_cat = batch_2019

Des terres |

cultivable | temp

s | 0 1 | Total

-----------+----------------------+----------

0 | 32 71 | 103

1 | 68 313 | 381

-----------+----------------------+----------

Total | 100 384 | 484

Pearson chi2(1) = 8.6447 Pr = 0.003

-------------------------------------------------------------------------------------------------------

-> ds_cat = batch_2020

Des terres |

cultivable | temp

s | 0 1 | Total

-----------+----------------------+----------

0 | 81 142 | 223

1 | 112 362 | 474

-----------+----------------------+----------

Total | 193 504 | 697

Pearson chi2(1) = 12.2050 Pr = 0.000

. bysort ds_cat: tab Q118Z_9 temp if year!=2021, chi2

-------------------------------------------------------------------------------------------------------

-> ds_cat = batch_2019

Une |

voiture ou |

une |

cammionnet | temp

te | 0 1 | Total

-----------+----------------------+----------

0 | 94 369 | 463

1 | 6 15 | 21

-----------+----------------------+----------

Total | 100 384 | 484

Pearson chi2(1) = 0.8380 Pr = 0.360

-------------------------------------------------------------------------------------------------------

-> ds_cat = batch_2020

Une |

voiture ou |

une |

cammionnet | temp

te | 0 1 | Total

-----------+----------------------+----------

0 | 178 477 | 655

1 | 15 27 | 42

-----------+----------------------+----------

Total | 193 504 | 697

Pearson chi2(1) = 1.4372 Pr = 0.231

. bysort ds_cat: tab Q118Z_10 temp if year!=2021, chi2

-------------------------------------------------------------------------------------------------------

-> ds_cat = batch_2019

| temp

Q118Z_10 | 0 1 | Total

-----------+----------------------+----------

0 | 48 150 | 198

1 | 52 234 | 286

-----------+----------------------+----------

Total | 100 384 | 484

Pearson chi2(1) = 2.6217 Pr = 0.105

-------------------------------------------------------------------------------------------------------

-> ds_cat = batch_2020

| temp

Q118Z_10 | 0 1 | Total

-----------+----------------------+----------

0 | 40 111 | 151

1 | 153 393 | 546

-----------+----------------------+----------

Total | 193 504 | 697

Pearson chi2(1) = 0.1386 Pr = 0.710

. bysort ds_cat: tab QH121 temp if year!=2021 & QH121!=998, chi2

-------------------------------------------------------------------------------------------------------

-> ds_cat = batch_2019

| temp

Pratique d'élévage | 0 1 | Total

----------------------+----------------------+----------

0 | 33 68 | 101

oui | 66 316 | 382

----------------------+----------------------+----------

Total | 99 384 | 483

Pearson chi2(1) = 11.6190 Pr = 0.001

-------------------------------------------------------------------------------------------------------

-> ds_cat = batch_2020

| temp

Pratique d'élévage | 0 1 | Total

----------------------+----------------------+----------

0 | 61 120 | 181

oui | 131 384 | 515

----------------------+----------------------+----------

Total | 192 504 | 696

Pearson chi2(1) = 4.5796 Pr = 0.032

1. **Test of difference between households surveyed once (lost to follow up) vs. twice in regard to the owning of livestock.**

. bysort ds_cat: ttest QH122B if year!=2021,by(temp)

-------------------------------------------------------------------------------------------------------

-> ds_cat = batch_2019

Two-sample t test with equal variances

------------------------------------------------------------------------------

Group | Obs Mean Std. err. Std. dev. [95% conf. interval]

---------+--------------------------------------------------------------------

0 | 99 .7676768 .3213134 3.197028 .1300409 1.405313

1 | 383 .7441253 .1325603 2.594257 .4834861 1.004765

---------+--------------------------------------------------------------------

Combined | 482 .7489627 .124136 2.725343 .5050469 .9928784

---------+--------------------------------------------------------------------

diff | .0235514 .3075934 -.5808444 .6279473

------------------------------------------------------------------------------

diff = mean(0) - mean(1) t = 0.0766

H0: diff = 0 Degrees of freedom = 480

Ha: diff < 0 Ha: diff != 0 Ha: diff > 0

Pr(T < t) = 0.5305 Pr(|T| > |t|) = 0.9390 Pr(T > t) = 0.4695

-------------------------------------------------------------------------------------------------------

-> ds_cat = batch_2020

Two-sample t test with equal variances

------------------------------------------------------------------------------

Group | Obs Mean Std. err. Std. dev. [95% conf. interval]

---------+--------------------------------------------------------------------

0 | 189 .9206349 .3048359 4.190801 .3192964 1.521973

1 | 501 1.938124 .3071931 6.875912 1.334575 2.541672

---------+--------------------------------------------------------------------

Combined | 690 1.65942 .2386789 6.269581 1.190795 2.128045

---------+--------------------------------------------------------------------

diff | -1.017489 .5341787 -2.066305 .0313272

------------------------------------------------------------------------------

diff = mean(0) - mean(1) t = -1.9048

H0: diff = 0 Degrees of freedom = 688

Ha: diff < 0 Ha: diff != 0 Ha: diff > 0

Pr(T < t) = 0.0286 Pr(|T| > |t|) = 0.0572 Pr(T > t) = 0.9714

. bysort ds_cat: ttest QH122C if year!=2021,by(temp)

-------------------------------------------------------------------------------------------------------

-> ds_cat = batch_2019

Two-sample t test with equal variances

------------------------------------------------------------------------------

Group | Obs Mean Std. err. Std. dev. [95% conf. interval]

---------+--------------------------------------------------------------------

0 | 99 .4848485 .1204366 1.198329 .245846 .723851

1 | 383 .7101828 .0808698 1.582653 .5511771 .8691885

---------+--------------------------------------------------------------------

Combined | 482 .6639004 .0689297 1.513317 .5284599 .799341

---------+--------------------------------------------------------------------

diff | -.2253343 .1704903 -.5603337 .1096652

------------------------------------------------------------------------------

diff = mean(0) - mean(1) t = -1.3217

H0: diff = 0 Degrees of freedom = 480

Ha: diff < 0 Ha: diff != 0 Ha: diff > 0

Pr(T < t) = 0.0935 Pr(|T| > |t|) = 0.1869 Pr(T > t) = 0.9065

-------------------------------------------------------------------------------------------------------

-> ds_cat = batch_2020

Two-sample t test with equal variances

------------------------------------------------------------------------------

Group | Obs Mean Std. err. Std. dev. [95% conf. interval]

---------+--------------------------------------------------------------------

0 | 192 .1875 .0421816 .5844853 .1042984 .2707016

1 | 504 .3888889 .038295 .8597214 .313651 .4641268

---------+--------------------------------------------------------------------

Combined | 696 .3333333 .0302511 .7980792 .2739388 .3927279

---------+--------------------------------------------------------------------

diff | -.2013889 .0672998 -.3335244 -.0692533

------------------------------------------------------------------------------

diff = mean(0) - mean(1) t = -2.9924

H0: diff = 0 Degrees of freedom = 694

Ha: diff < 0 Ha: diff != 0 Ha: diff > 0

Pr(T < t) = 0.0014 Pr(|T| > |t|) = 0.0029 Pr(T > t) = 0.9986

. bysort ds_cat: ttest QH122D if year!=2021,by(temp)

-------------------------------------------------------------------------------------------------------

-> ds_cat = batch_2019

Two-sample t test with equal variances

------------------------------------------------------------------------------

Group | Obs Mean Std. err. Std. dev. [95% conf. interval]

---------+--------------------------------------------------------------------

0 | 99 2.393939 .4436543 4.414305 1.513522 3.274357

1 | 383 3.793734 .3265644 6.390992 3.151645 4.435823

---------+--------------------------------------------------------------------

Combined | 482 3.506224 .2760444 6.060417 2.963822 4.048626

---------+--------------------------------------------------------------------

diff | -1.399794 .6810171 -2.737937 -.0616512

------------------------------------------------------------------------------

diff = mean(0) - mean(1) t = -2.0554

H0: diff = 0 Degrees of freedom = 480

Ha: diff < 0 Ha: diff != 0 Ha: diff > 0

Pr(T < t) = 0.0202 Pr(|T| > |t|) = 0.0404 Pr(T > t) = 0.9798

-------------------------------------------------------------------------------------------------------

-> ds_cat = batch_2020

Two-sample t test with equal variances

------------------------------------------------------------------------------

Group | Obs Mean Std. err. Std. dev. [95% conf. interval]

---------+--------------------------------------------------------------------

0 | 192 2.703125 .4511498 6.251315 1.813249 3.593001

1 | 504 3.162698 .2439642 5.476982 2.683384 3.642013

---------+--------------------------------------------------------------------

Combined | 696 3.03592 .2160654 5.700196 2.6117 3.460139

---------+--------------------------------------------------------------------

diff | -.4595734 .4834579 -1.408789 .489642

------------------------------------------------------------------------------

diff = mean(0) - mean(1) t = -0.9506

H0: diff = 0 Degrees of freedom = 694

Ha: diff < 0 Ha: diff != 0 Ha: diff > 0

Pr(T < t) = 0.1711 Pr(|T| > |t|) = 0.3421 Pr(T > t) = 0.8289

. bysort ds_cat: ttest QH122E if year!=2021,by(temp)

-------------------------------------------------------------------------------------------------------

-> ds_cat = batch_2019

Two-sample t test with equal variances

------------------------------------------------------------------------------

Group | Obs Mean Std. err. Std. dev. [95% conf. interval]

---------+--------------------------------------------------------------------

0 | 99 1.636364 .3306804 3.290228 .9801392 2.292588

1 | 383 3.386423 .3949603 7.729526 2.609855 4.162991

---------+--------------------------------------------------------------------

Combined | 482 3.026971 .3225778 7.082033 2.393135 3.660807

---------+--------------------------------------------------------------------

diff | -1.750059 .7953108 -3.31278 -.1873384

------------------------------------------------------------------------------

diff = mean(0) - mean(1) t = -2.2005

H0: diff = 0 Degrees of freedom = 480

Ha: diff < 0 Ha: diff != 0 Ha: diff > 0

Pr(T < t) = 0.0141 Pr(|T| > |t|) = 0.0282 Pr(T > t) = 0.9859

-------------------------------------------------------------------------------------------------------

-> ds_cat = batch_2020

Two-sample t test with equal variances

------------------------------------------------------------------------------

Group | Obs Mean Std. err. Std. dev. [95% conf. interval]

---------+--------------------------------------------------------------------

0 | 191 2.198953 .4469806 6.177395 1.317271 3.080635

1 | 504 2.831349 .2649835 5.948865 2.310738 3.35196

---------+--------------------------------------------------------------------

Combined | 695 2.657554 .2281502 6.014691 2.209606 3.105501

---------+--------------------------------------------------------------------

diff | -.6323963 .5108663 -1.635428 .3706351

------------------------------------------------------------------------------

diff = mean(0) - mean(1) t = -1.2379

H0: diff = 0 Degrees of freedom = 693

Ha: diff < 0 Ha: diff != 0 Ha: diff > 0

Pr(T < t) = 0.1081 Pr(|T| > |t|) = 0.2162 Pr(T > t) = 0.8919

. bysort ds_cat: ttest QH122F if year!=2021,by(temp)

-------------------------------------------------------------------------------------------------------

-> ds_cat = batch_2019

Two-sample t test with equal variances

------------------------------------------------------------------------------

Group | Obs Mean Std. err. Std. dev. [95% conf. interval]

---------+--------------------------------------------------------------------

0 | 99 4.959596 .7174417 7.138455 3.535856 6.383336

1 | 383 6.464752 .5731954 11.21766 5.337739 7.591765

---------+--------------------------------------------------------------------

Combined | 482 6.155602 .4792128 10.52088 5.213993 7.097211

---------+--------------------------------------------------------------------

diff | -1.505156 1.185447 -3.834463 .8241515

------------------------------------------------------------------------------

diff = mean(0) - mean(1) t = -1.2697

H0: diff = 0 Degrees of freedom = 480

Ha: diff < 0 Ha: diff != 0 Ha: diff > 0

Pr(T < t) = 0.1024 Pr(|T| > |t|) = 0.2048 Pr(T > t) = 0.8976

-------------------------------------------------------------------------------------------------------

-> ds_cat = batch_2020

Two-sample t test with equal variances

------------------------------------------------------------------------------

Group | Obs Mean Std. err. Std. dev. [95% conf. interval]

---------+--------------------------------------------------------------------

0 | 190 7.036842 .6373841 8.785733 5.779541 8.294143

1 | 503 8.88668 .5770754 12.94245 7.752899 10.02046

---------+--------------------------------------------------------------------

Combined | 693 8.379509 .4547 11.96993 7.486752 9.272266

---------+--------------------------------------------------------------------

diff | -1.849838 1.017596 -3.847789 .1481132

------------------------------------------------------------------------------

diff = mean(0) - mean(1) t = -1.8179

H0: diff = 0 Degrees of freedom = 691

Ha: diff < 0 Ha: diff != 0 Ha: diff > 0

Pr(T < t) = 0.0348 Pr(|T| > |t|) = 0.0695 Pr(T > t) = 0.9652
