# Supplementary figures and images for: COVID-19 and its effects on food producers: panel data evidence from Burkina Faso
Source: BMC Nutr. 2024 Oct 8;10:132. doi: 10.1186/s40795-024-00942-x (PMC11462756; doi:10.1186/s40795-024-00942-x)

Appendix 2


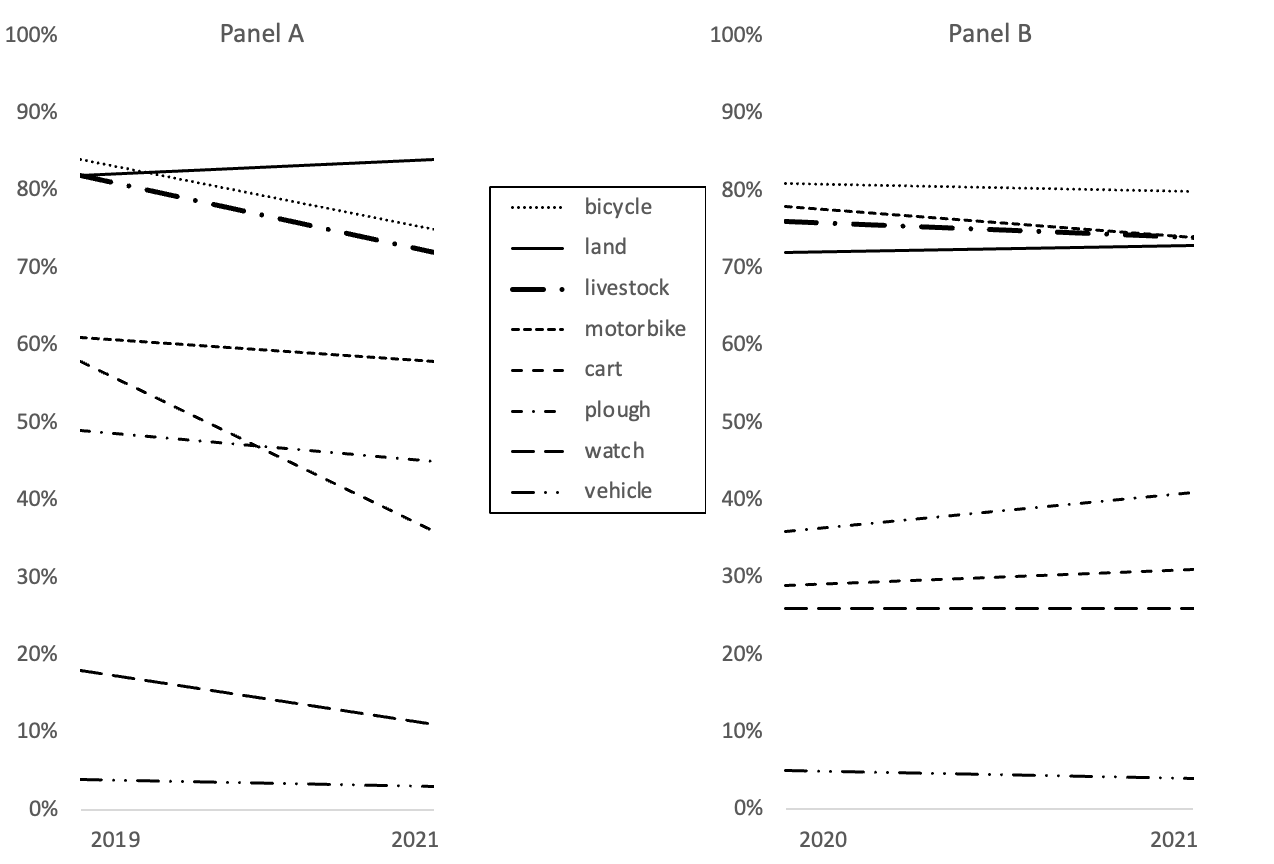

Supplement: Supplementary file 2 — Supplementary Material 2 [file 40795_2024_942_MOESM2_ESM.docx]
